# Crack Size and Undermatching Effects on Fracture Behavior of a Welded Joint

**DOI:** 10.3390/ma16134858

**Published:** 2023-07-06

**Authors:** Aleksandar Sedmak, Elisaveta Doncheva, Bojan Medjo, Marko Rakin, Nenad Milosevic, Dorin Radu

**Affiliations:** 1 Faculty of Mechanical Engineering, University of Belgrade, Kraljice Marije 16, 11120 Belgrade, Serbia; 2 Faculty of Civil Engineering, Transilvania University of Brașov, Turnului Street 5, 500152 Brașov, Romania; 3 Faculty of Mechanical Engineering, University of Skopje, Boulevard 8-mi Septemvri, 1000 Skopje, North Macedonia; 4 Faculty of Technology and Metallurgy, University of Belgrade, Karnegijeva 4, 11120 Belgrade, Serbia

**Keywords:** welded joint mismatching, crack tip fields, finite element method

## Abstract

Crack size and undermatching effects on fracture behavior of undermatched welded joints are presented and analyzed. Experimental and numerical analysis of the fracture behavior of high-strength low-alloyed (HSLA) steel welded joints with so-called small and large crack in undermatched weld metal and the base metal was performed, as a part of more extensive research previously conducted. J integral was determined by direct measurement using special instrumentation including strain gauges and a CMOD measuring device. Numerical analysis was performed by 3D finite element method (FEM) with different tensile properties in BM and WM. Results of J-CMOD curves evaluation for SUMITEN SM 80P HSLA steel and its weld metal (WM) are presented and analyzed for small and large cracks in tensile panels. This paper is focused on some new numerical results and observations on crack tip fields and constraint effects of undermatching and crack size keeping in mind previously performed experiments on the full-scale prototype. In this way, a unique combined approach of experimental investigation on the full-scale proto-type and tensile panels, as well as numerical investigation on mismatching and crack size effects, is achieved.

## 1. Introduction

Welded joint heterogeneity has an important role in the behavior of steel welded joints, particularly if crack-like defects are present, causing local plastic strains. Even in the case of filler metal being the same class as the base metal, a welded joint has different tensile properties, toughness, fracture toughness, and fatigue crack growth rate as a consequence of heterogeneous microstructure, at least in four zones of the joint (base metal—BM, weld metal—WM, coarse-grain heat-affected zone—CGHAZ, fine-grain heat-affected zone—FGHAZ), [1,2,3,4,5,6,7,8,9,10,11]. Different tensile properties are analyzed and evaluated in recent papers [1,2,3], where the digital image correlation (DIC) technique was used to measure strains, and the finite element method (FEM) was used to calculate stress distribution in specimens with a rectangular cross-section to evaluate true stress–strain curves more precisely. The effect of material heterogeneity on tensile properties and fracture toughness is presented in paper [4], indicating WM as the weakest zone of welded joint made of SUMITEN SM 80P HSLA steel, while different aspects of fracture toughness were analyzed for welded joints, made of different HSLA steels, and presented in papers [5,6,7,8,9]. Charpy toughness and fracture toughness in different zones of a welded joint were analyzed in paper [10], indicating a strong effect of material heterogeneity and HAZ as the weakest link in SUMITEN SM 80P HSLA steel. Also, fatigue crack growth rate in different zones of two HSLA steel welded joints was evaluated experimentally by using the Paris law, as presented in papers [11,12].

Fracture behavior of cracked undermatched welded joints made of HSLA steel was analyzed and presented in number of papers, where so-called strength mismatching was defined as the ratio between WM and BM yield strength (YS). In [13,14], HSLA steel in a quenched and tempered condition, corresponding to the grade HT 80, was investigated. The flux cored arc welding process (FCAW), with CO_2_ as shielding gas, was used and two different tubular wires were selected as filler metals. Three differently undermatched welded joints were analyzed using results of testing the notched specimens with through-thickness crack front positioned partly in WM, partly in HAZ, and partly in BM. It was shown that the presence of different microstructures along the pre-crack fatigue front had an important effect on the critical crack tip opening displacement (CTOD), indicating that the fracture behavior strongly depends on the proportion of ductile base material, as well as on the size and distribution of the mismatching factor along the vicinity of crack front. In paper [15], the fracture mechanics analysis of specimens, with surface notch tips completely embedded in the heat affected zones, was performed. The results showed that the strength of mismatching of a welded joint caused a redirection of the crack propagation towards the low-strength region of the welded joint. It was also shown that even in the case of overmatched welded joints, but with a soft root layer, it was possible to achieve satisfying crack resistance, proving that such a type of welded joint is preferable for the welding of HSLA steels, because it enables the manufacturing of a welded joint without preheating.

More recently, full-scale experimental investigation was conducted on welding joints made of APL X80 wide plates [16]. Tensile tests were performed on Ø1422 mm × 25.7 mm X80 pipeline with original and repaired welding joints, equipped with strain gauges and using digital image correlation (DIC) method to measure strains and evaluate difference in loading capacity. In paper [17], effects of multiple defects on an overmatched welded joint fracture behavior under static loading were investigated numerically, by FEM, and experimentally, by DIC. It was shown that even in the case of a ductile structural steel (S235), fracture can occur at a relatively low stress level. Another study with DIC was performed to obtain the strain distribution in undermatching X80 pipe weld joints under uniaxial tensile loading, [18]. The results showed that the maximum strain was in the WM.

The yield strength mismatch in X80 pipeline steel welds, obtained by gas metal arc welding (GMAW) process, was estimated using instrumented indentation [19]. All three different levels of WM yield strengths (even, over, and undermatched) were investigated. In [20], a method for testing the local properties of girth welded joints in pipelines is proposed based on DIC measurement to identify the true stress–strain curves and local mechanical properties. Also, FEM, based on the GTN model, was used to verify the local mechanical properties of girth welded joints obtained by using DIC.

The focus here is on crack size and undermatching effects on fracture behavior of a welded joint of SUMITEN SM 80P HSLA steel. From a design point of view, strength overmatching is preferable, so that weld metal (WM) has higher YS compared to base metal (BM), but this is not always good idea from a structural integrity point of view, as explained in papers [21,22]. As a general rule, HSLA steel’s sensitivity to cracking increases with increasing level of strength, so an undermatching effect is a more likely design solution for YS above 700 MPa [21,22]. Anyhow, it is not as simple as just avoiding cracking, since eventual plastic strain (due to stress concentration and low YS) would be localized in the weld metal until its strain hardening capacity is partly or fully exhausted before base metal would even start to yield [21,22,23,24].

**Figure 1:**
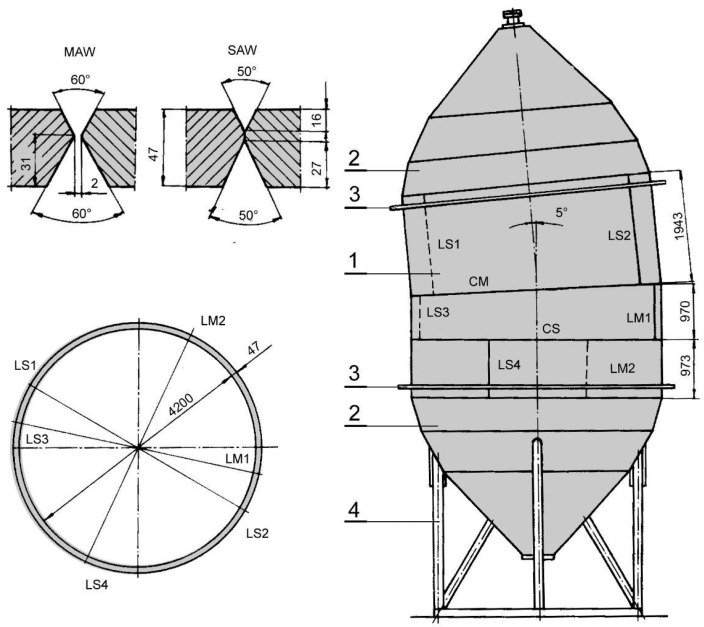
The full-scale model: 1—mantle; 2—lid; 3—stiffener; 4—supports. L—longitudinal, C—circular welded joint; MAW—manual arc welding (M); SAW—submerged arc welding (S) [21,22].

In welded pressure vessels, stress concentrations caused by geometrical changes, including inevitable weldment imperfections, such as angular distortion or misalignment, can produce local plastic strains, possibly exhausting a portion of the strain hardening capacity. In these circumstances, the question arises of how cracks would behave [21,22,23,24]. As an example of such a problem, one can use the penstock in Reversible Hydro Power Plant “BAJINA BASTA” (RHPP BB), designed with a reduced safety factor [21,22] to fulfill the basic requirement—to make one instead of two penstocks. Consequently, HSLA steel was used, SUMITEN 80P, with YS around 700 MPa, but only after extensive experimental research of the prototype, as shown in Figure 1, to prove its fitness-for-purpose, as described in [21,22]. Later on, this approach was named the structural integrity [25]. Although this issue was a topic of a number of papers decades ago, only recently some of the most intriguing results have been explained in detail by using the finite element method for precise analysis of the stress–strain state, both for the prototype [26,27,28] and for tensile panels with large and small cracks, as shown in [29,30] for the BM. Here, the attention is focused on some new numerical results for the WM and observations on crack tip fields and constraint effects due to undermatching and crack size effects, obtained by comparison with the previous results for the BM. The novelty in this approach is the unique combination of experimental investigation on the full-scale prototype and tensile panels, as well as numerical investigation of mismatching and crack size effects in the case of an undermatched welded joint with a crack in the weld metal. A similar approach was applied in [6,31,32] but focused on cracks in HAZ and constraint effects. Mismatching and constraint effects in a different HSLA steel (Niomol 490) with differently positioned cracks in weld metal were analyzed in [33] by using a micromechanical approach to simulate crack growth. Such an approach requires determination of parameters, which are beyond the scope of this investigation but could be of interest for a future work.

## 2. Materials and Methods

### 2.1. Base Metal and Welding

The base metal in this research was SUMITEN SM 80P HSLA steel produced in Japan, used for construction of a large penstock in RHPP BB in Serbia, as well as for the full-scale prototype, as shown in Figure 1. Chemical composition of BM and WMs is given in Table 1, while the tensile properties are given in Table 2, indicating undermatched welded joint both in the case of shielded manual arc welding (SMAW) and submerged arc welding (SAW), which were used alternatively to produce the full-scale prototype. The mismatching ratio was 0.91 for SAW and 0.95 for SMAW, being in accordance with the high YS of the BM.

Both welding processes, SMAW and SAW, were used for penstock welding, and also applied under the same conditions to produce the full-scale prototype, which was used for extensive testing to prove fitness-for-service, [21,22]. The basic coated low-hydrogen electrode LB 118 for MAW and core wire US 8013 with M38F flux for SAW welding, produced by “Kobe Steel”, Kobe, Japan, were used. Post-weld heat treatment was applied to release residual stresses.

### 2.2. Tensile Panels

**Figure 2:**
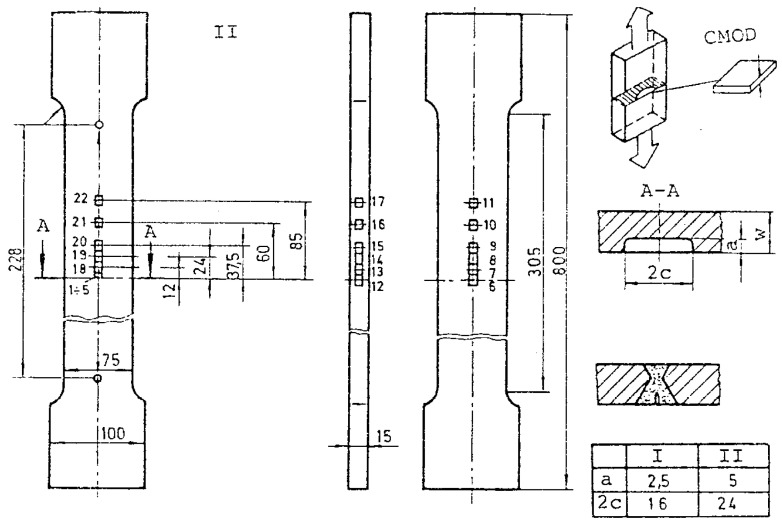
Pre-cracked tensile panels with details of surface cracks [21,22].

Tensile panels (TP) were made from the base metal (SM 80P) and also from welded joints of different mismatching levels, with the so-called large surface crack (LSC), 5 × 24 mm, and small surface crack (SSC), 2.5 × 16 mm, as shown in Figure 2. They were tested in the scope of fitness-for-service experimental investigation, to obtain better insight of mismatching effects on stress–strain behavior, as shown in [21,22].

### 2.3. Numerical Analysis—FEM

**Figure 3:**
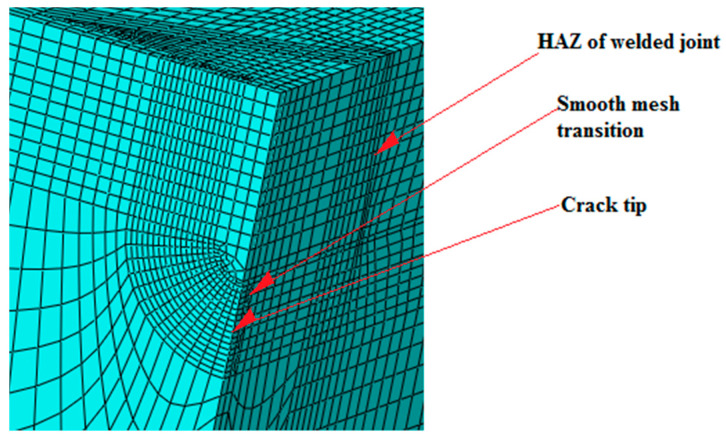
Details of surface small crack in weld metal [30].

Three-dimensional FE models were developed to simulate behavior of tensile panels with SSC and LSC. The effect of the crack tip fields, mismatching, and constrains were carefully studied using Abaqus, as described in [29,30,34]. Base and weld metal were assumed to behave in an isotropic elastic–plastic manner. The finite element mesh was made of regular elements and refined in the vicinity of the crack tip with 0.2 × 0.2 mm elements. As an example, such a mesh is shown in Figure 3 for TP with SSC. Crack growth was not simulated, i.e., the analysis was performed for stationary cracks.

**Figure 4:**
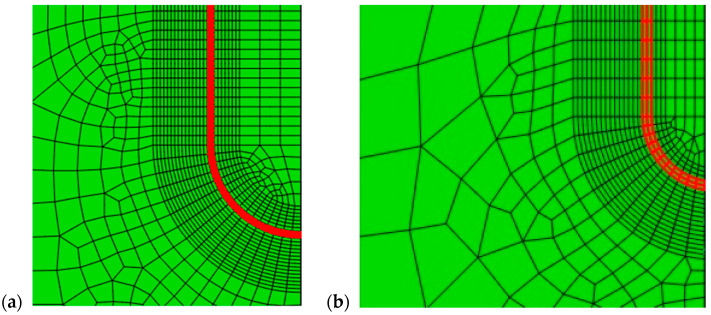
Geometry of numerically representation: (**a**) LSC, (**b**) SSC. Red lines mark crack front.

Only a quarter of the specimen is modeled due to symmetry conditions. The 20-node quadratic isoparametric elements, C3D20R, were used—26,932 of them for TP with SSC in weld metal (WM SSC model) and 19,176 for TP with LSC in weld metal (WM LSC model). Some details of the FE mesh for TP with LSC and SSC are shown in Figure 4.

The CMOD is obtained by tracking the positions of the two nodes located at the crack mouth, while the values of the J integral are obtained by the domain integral method. The domain was sufficiently distant from the crack front to ensure the convergence of the J integral values.

### 2.4. Stress–Strain Curves

The most intriguing part of FEM simulation is how to evaluate stress–strain curves for all zones of a welded joint (BM, WM, and HAZ, with both coarse and fine grain subzones). It was shown in [1,2,3,9] how it can be done for true stress–strain curves, based on the iteration procedure originally introduced for engineering stress–strain curves in [35,36]. In the case analyzed here, a slightly simplified procedure was adopted, since the stress–strain curves were evaluated for BM and WM only, having in mind that the mismatching between BM and WM was in our focus, so the effect of HAZ and its subzones was neglected. Both cracks, SSC and LSC, positioned in WM, grew only through WM, i.e., they did not enter into the HAZ, and the same holds for the BM, which means that bi-material modeling approach can be applied.

**Figure 5:**
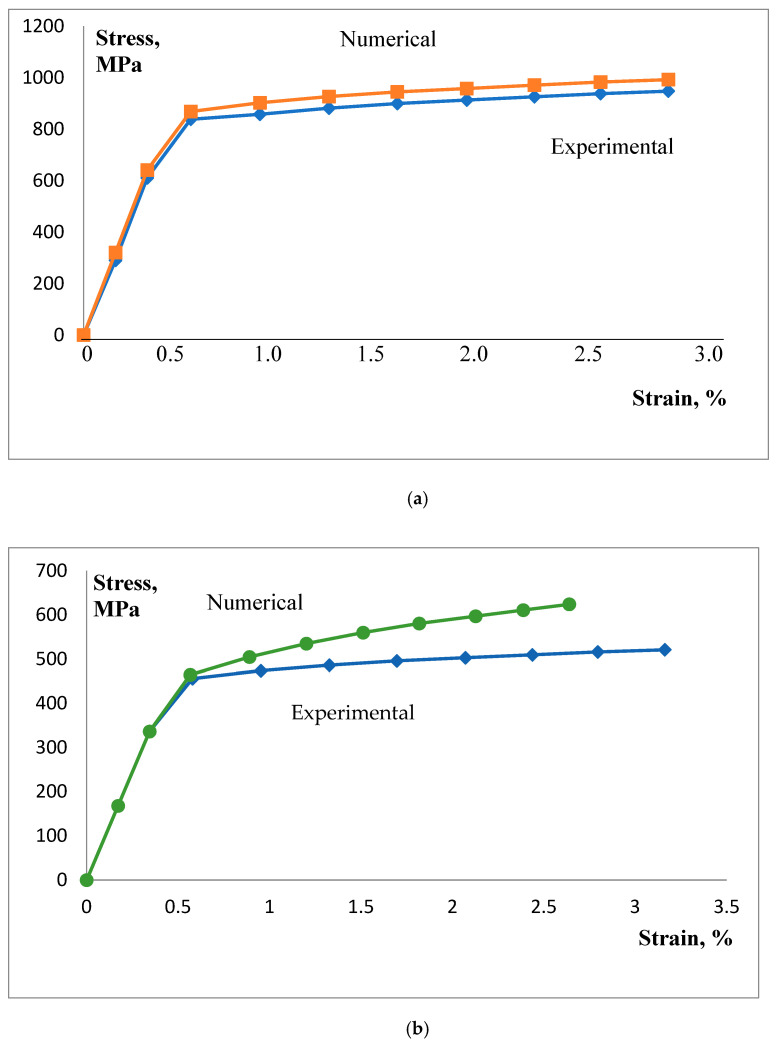
True stress–true strain curves. (**a**) Base metal; (**b**) weld metal.

True stress–strain curves, as used in this research, are shown in Figure 5, indicating better agreement between numerical and experimental values for BM than for WM.

## 3. Numerical Results of BM and WM with SSC and LSC

### Von Mises Stress Distribution

**Figure 6:**
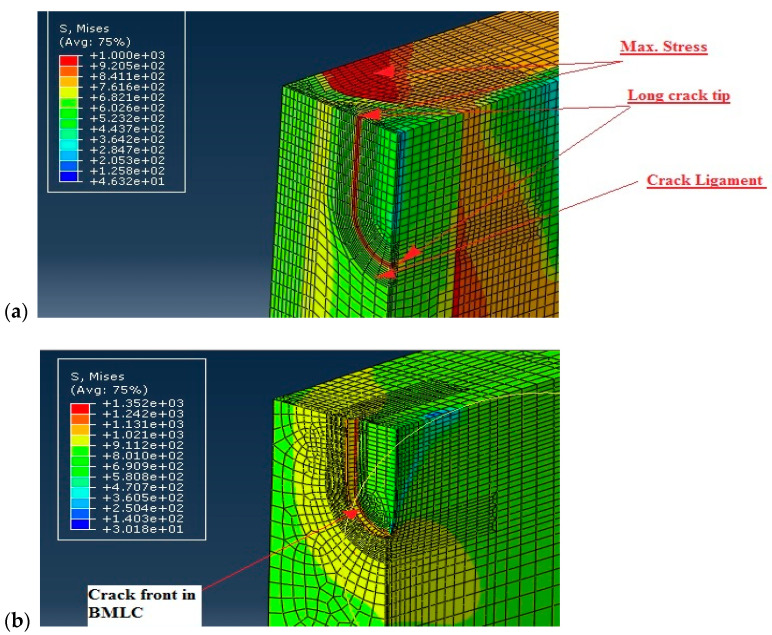
Comparison of the effects of LSC tip stress fields in (**a**) WM, (**b**) BM. Stresses are given in MPa.

**Figure 7:**
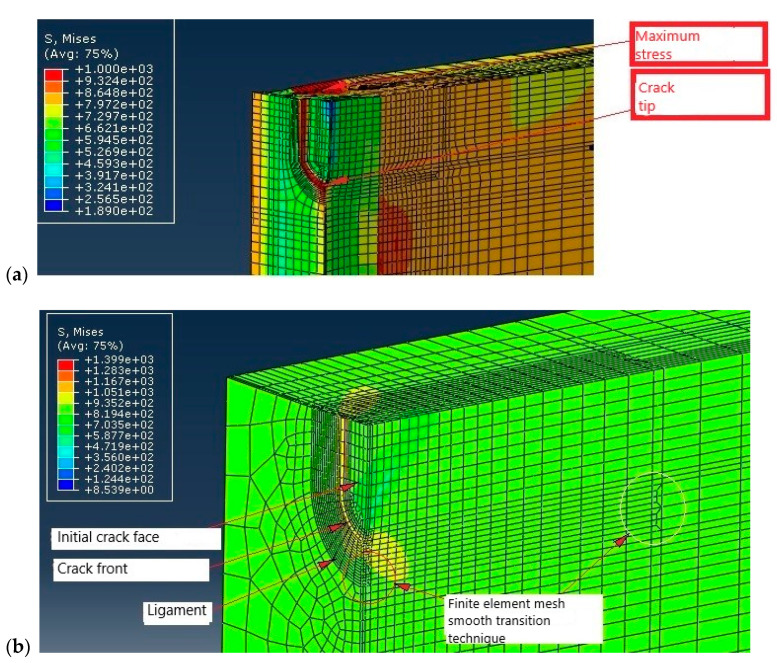
Comparison of the effects of SSC tip stress fields in (**a**) WM, (**b**) BM. Stresses are given in MPa.

Figure 6 shows distribution of von Mises stress in the WM for LSC (Figure 6a) and BM for LSC (Figure 6b), whereas Figure 7 shows its distribution for SSC in the same way.

**Figure 8:**
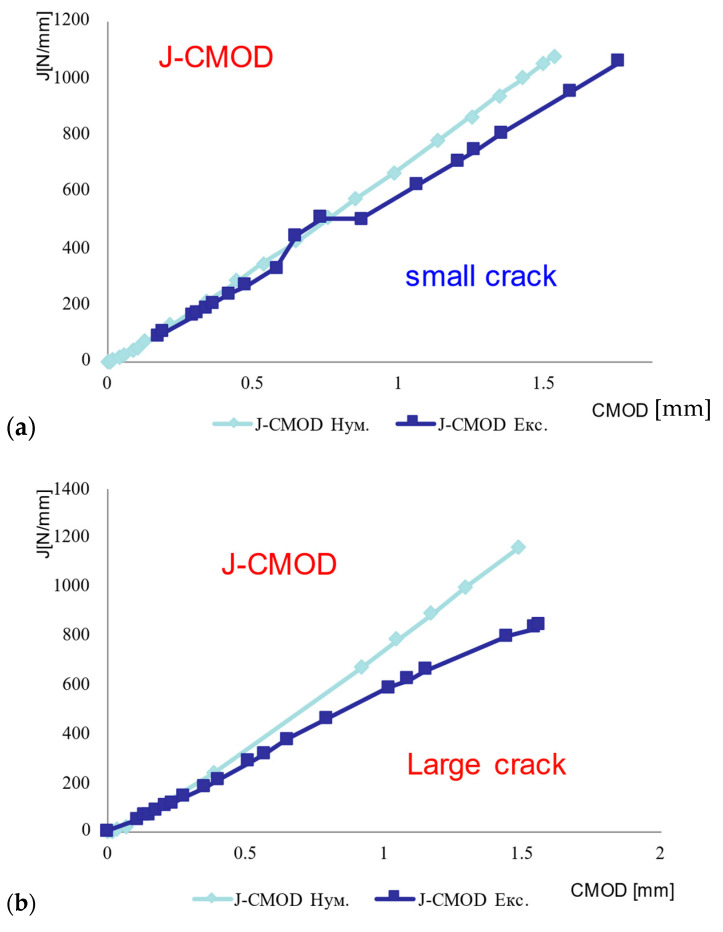
J-CMOD curves: (**a**) WM SSC, (**b**) WM LSC.

**Figure 9:**
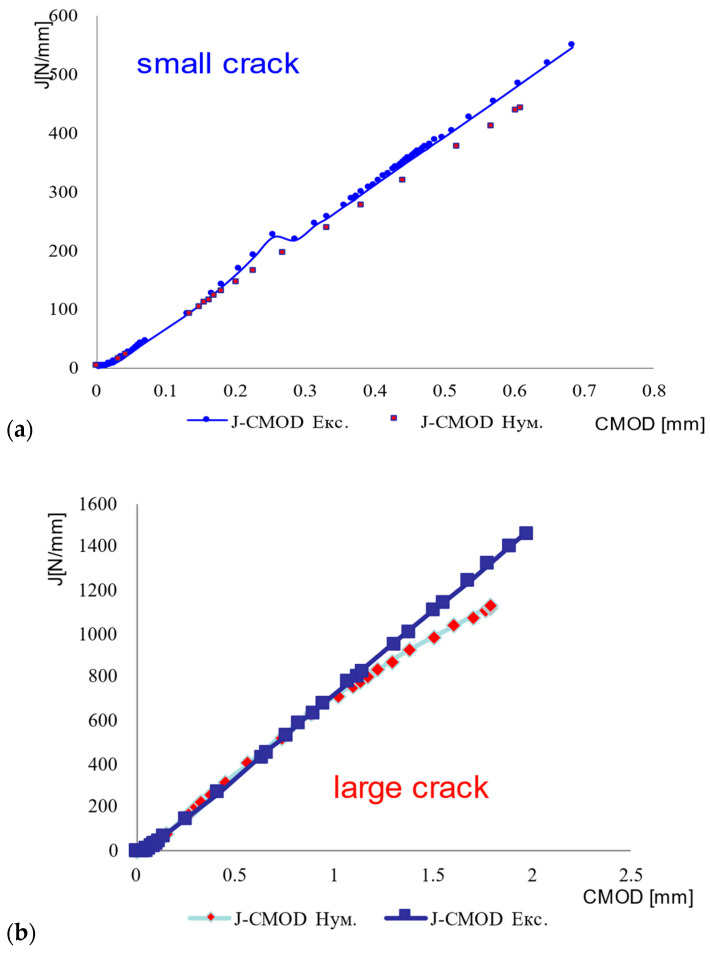
J-CMOD curves: (**a**) BM SSC, (**b**) BM LSC.

Based on the procedure for CMOD and J calculations described in #2.3, the J-CMOD curves are obtained for BM SSC and WM SSC, Figure 8, as well as BM LSC and WM LSC, Figure 9. One can see that differences between experimental and numerical values increase with increasing J, which is a consequence of numerical modeling without taking crack growth into account. Anyhow, crack growth was not in focus of this research.

## 4. Discussion

Fracture behavior of the undermatched welded joint was analyzed regarding the effect of BM and WM mismatching on the crack tip fields, as well as the effect of crack size (SSC vs. LSC). Figure 6 shows significant difference in stress distribution around LSC due to the mismatching effect, as shown in Figure 6a (WM) compared with Figure 6b (BM). Namely, contrary to the BM, where the maximum stress is located at the crack tip, maximum stress in the undermatched welded joint appears both at the crack tip in the WM and in the BM, next to it. Such a redistribution of stresses is beneficial for welded joint resistance to cracking, since it provides reduced crack driving force in WM.

The same comparison can be made for the SSC, Figure 7. As one can see from Figure 7a, the maximum stress in the undermatched welded joint appears both at the crack tip in the WMand in the BM, next to it, whereas the maximum stress in the BM is located at the crack tip. Such a redistribution of stresses indicates more favorable fracture behavior of the WM, as in the case of LSC. One should notice that in both cases, SSC and LSC, the beneficial effect of mismatching is possible only if the WM is capable of sustaining at least small amount of plastic strain. This condition is fulfilled in the analyzed case, as shown in Table 2, since the WM elongation is at least 22%.

On the other side, one can see from Figure 6 and Figure 7 that differences in stress fields for the LSC and SSC are not significant, both for the WM and BM. In the case of WM, the maximum stress is the same, while in the case of BM, maximum stress is somewhat higher for the SSC compared to LSC. Obviously, the effect of mismatching is dominant in the case analyzed here.

As one can see from Figure 8a, experimental and numerical values for maximum J in the case of WM with SSC are at the same level, cca. 1000 N/mm, with a small difference for maximum CMOD values (experimental value 1.8 mm, numerical 1.6 mm), probably due to pop-in effect. In the case of WM with LSC, numerical and experimental results for maximum CMOD value agree well, but maximum J value is significantly higher when calculated. One can also notice from both Figure 8a,b that FEM values for CMOD (at the same level of J) are consistently lower than the experimental ones, probably due to crack growth effects, as already mentioned. Obviously, for a shorter crack one obtains smaller values for CMOD. Nevertheless, the differences are not significant.

Agreement between experimental and numerical results is better in the case of the BM, Figure 9, which was expected since the modeling of BM tensile behavior is simpler and thus more precise than the modeling of WM, as already shown in Figure 5. One should notice that in the case of BM, both for SSC and LSC, numerical CMOD values are higher than experimental ones for the same level of J integral, contrary to the WM behavior. Obviously, crack growth does not play an important role in the case of BM, as also shown in [5], indicating only 1 mm of crack growth, compared with more than 5 mm in the case of WM.

From Figure 8 and Figure 9 it is also clear that the agreement between experimental and numerical results is better for BM than for WM, as one could expect due to better agreement of BM stress–strain curves than of WM ones, Figure 5.

Another important aspect of fracture behavior of undermatched welded joint is the comparison with overmatching effect, which was analyzed and presented in [37] in two cases—the crack tip positioned in the course-grain (CG) HAZ and in the fine-grain (FG) HAZ. For both cases, it was shown that the overmatching effect was beneficial for the overall welded joint resistance to crack growth, even though local crack growth was promoted by a high tri-axial stress state in the case of crack tip in the FG HAZ.

One should notice that in both cases, under- and overmatching effects are favorable for fracture behavior since BM acts as barrier to crack growth. Actually, heterogeneity in this case is beneficial since welded joint behaves better then WM and/or HAZ would behave as homogeneous structures.

## 5. Conclusions

Experimental and numerical methods have been used to characterize fracture behavior of undermatched welded joints, made of HSLA steel SM 80P. Based on this research, the following conclusions are obtained:

- Mismatching effects play a more significant role in fracture behavior of undermatched welded joints than crack size, since the crack tip fields are influenced mostly by mismatching, and to smaller a extent, by crack size.
- Crack tip fields in the case of an undermatched welded joint are favorable, since high stresses are re-distributed from the crack tip to the BM.
- Numerical results agree well with the experimental ones, with increasing differences in the case of WM due to crack growth, which was not taken into account in numerical modeling.
- Differences between numerical and experimental results in the case of WM are larger than in the case of BM, which is attributed to the modeling of stress–strain curves, being less precise in the case of WM.

## Figures and Tables

**Table 1:** Chemical composition of SM 80P steel and of MAW and SAW weld metals.

Element		C	Si	Mn	P	S	Cu	Cr	Ni	Mo	V	B	C_eq_
SM 80P		0.10	0.30	0.90	0.01	0.008	0.24	0.48	1.01	0.47	0.03	0.0016	0.5
Weld metal	MAW	0.06	0.53	1.48	0.011	0.005	-	0.24	1.80	0.43	-	-	-
	SAW	0.07	0.37	1.87	0.01	0.011	-	0.44	0.13	0.73	-	-	-

**Table 2:** Tensile properties of SM 80P steel and of weld metal for SMAW and SAW.

Material	Direction	Tensile Properties		
		Y.S. [MPa]	U.T.S. (MPa)	Elongation (%)
SM 80P	Rolling	Min. 755	Min. 804	Min. 24
	cross rolling	Min. 755	Min. 795	Min. 22
Weld metal	SMAW	722	810	22
	SAW	687	804	23

## Data Availability

Not applicable.
